# Recognizing and stabilizing miR-21 by chiral ruthenium(II) complexes

**DOI:** 10.1186/s13065-020-00672-8

**Published:** 2020-04-03

**Authors:** Yin Feng, Jing Shu, Liangzhong Yao, Yutao Lan, Lianbao Ye, Wenjie Mei, Ying Ding

**Affiliations:** 1 The First Affiliated Hospital of Guangdong Pharmaceutical University, Guangzhou, 510062 China; 2 School of Pharmacy, Guangdong Pharmaceutical University, Guangzhou, 510006 China; 3 Guangdong Province Engineering Center for Molecular Probe & Biomedical Imaging, Guangzhou, 510006 China; 4 School of Nursing, Guangdong Pharmaceutical University, Guangzhou, Guangdong 510006 China; 5 Guangzhou Key Laboratory of Construction and Application of New Drug Screening Model System, Guangdong Pharmaceutical University, Guangzhou, 510006 China

**Keywords:** Chiral ruthenium(II) complexes, MiR-21, RNA binding property, FRET

## Abstract

MiR-21, a non-coding miRNA with 22 nucleotides, plays an important part in the proliferation, invasion, and metastasis of tumor cells. The present study demonstrates that isomers of chiral ruthenium(II) complexes with alkynes (Λ-1 and Δ-1) were synthesized by Songogashira coupling reaction by using microwave-assisted synthetic technology. The isomers can recognize and stabilize miR-21, with the Λ-isomer showing a stronger binding capacity than the Δ-isomer. Further studies showed that both isomers can be uptaken by MDA-MB-231 cells and enriched in the nucleus. Treatment with the Λ-/Δ-isomer downregulated the expression of miR-21. In a word, the development of chiral ruthenium(II) complexes act as potential inhibitors against tumor cells by recognizing, stabilizing, and regulating the expression of miR-21.

## Introduction

**Fig. 1:**
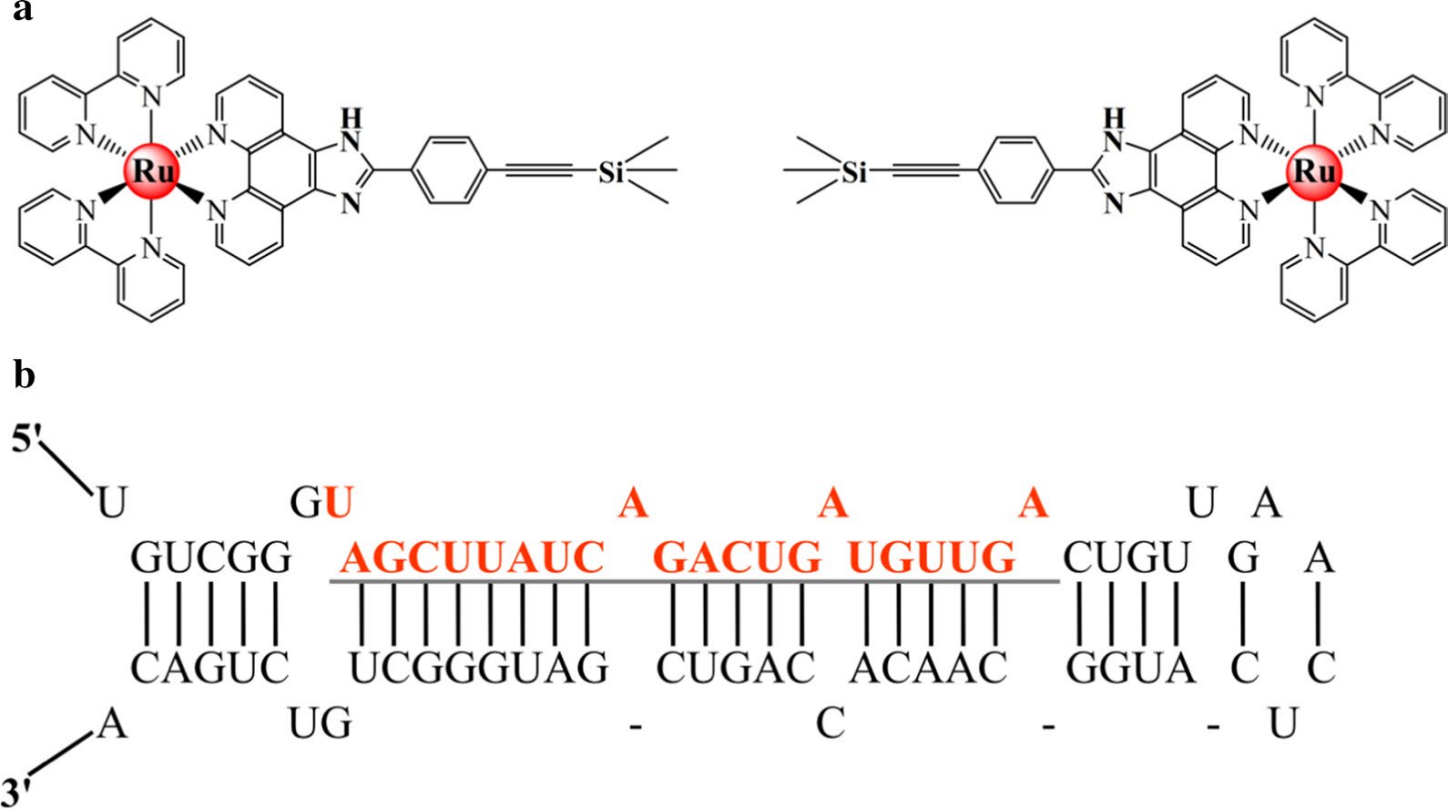
**a** Molecular structures of the chiral ruthenium(II) complexes ***Λ****/****Δ***-1. **b** Structure of pre-miR-21 and mature miR-21 (Red)

Increasing attention has been focused on small-molecule targeting drugs over the last decades, and some of these drugs, including NAMI-A, KP1019, and CX-3543, have been undergone clinical trials [1–5]. However, with the rapid development of tumor targeting small molecules, the multidrug resistance and dependencies of cancer cells have become more serious [6–8]. Therefore, novel compounds that suppress the proliferation of tumor cells by regulating the expression of target genetic fragments must be designed [9]. MicroRNAs (miRNAs), a type of endogenous non-coding RNAs molecules with ~ 22 nucleotides that regulate the expression of protein by cleaving or repressing the translation of target mRNAs [10–14], provide the opportunities [15, 16]. In the last decades, more than 1000 miRNAs have been found in human cells; some of these miRNAs are highly tissue-specific expression, whereas others are identified as house-keeping functional molecules [17–19]. MiR-21 (Fig. 1b), a miRNA overexpressed in almost all types of human malignancy, is involved in multifarious cancer-associated processes, including proliferation, invasion, and metastasis [20–22]. A growing number of evidence suggests that miR-21 as an “oncomir” in oncogenesis, which is up-regulation in most detected cancers, including breast, colorectal, pancreatic and glioblastoma cancer [23–25]. In recent years, miR-21 has been investigated as a potent target for small-molecule drugs [26, 27]. For example, the gemcitabine sensitivity of cholangiocarcinoma cells increases by suppressing miR-21 [28]. In addition, curcumin could suppress tumor cells growth, invasion and metastasis by significantly inhibiting miR-21 expression [29]. A similar phenomenon was also observed in xenograft mouse models of gliomas, human hepatocytes, and breast cancer cells [30]. A recent study has found that miR-21 regulates cell apoptosis. Sasaki indicated that the apoptosis induction of tumor cells can be suppressed by upregulating the expression of miR-21 [31]. Further studies showed that overexpressing miR-21 can regulate the level of B-cell lymphoma-2 (Bcl-2) protein by downregulating Bcl-2 and upregulating B-cell lymphoma-associated x [32]. In addition, suppressing the expression of miR-21 activates the caspase signal pathway and downregulates the expression of caspase-3 [33]. Overexpressing miR-21 can inhibit the expression of the P53 gene, which exerts proapoptotic effect [34]. It can also repress the apoptosis of cells by regulating the programmed cell death 4 gene [35].

Recently, Ruthenium(II) complexes have been extensively studied as potential anti-tumor agents [36–40]. Many studies showed that ruthenium(II) complexes containing planar aromatic rings can interact with DNA molecules through intercalative binding, groove-binding, and/or electrostatic interaction mode, and some entities with strong inhibitory effect have been researched [41–43]. Ruthenium complexes bearing different ligands could also bind and stabilize G-quadruplex DNA, including telomeric and proto-oncogene, which display great inhibition against tumor cells by regulating the expression of related proteins [44–48]. In our previous study found that polypyridyl ruthenium(II) complexes induced apoptosis through a mitochondria-mediated pathway rather than by direct-interaction DNA damage [49]. Further study suggested that chiral ruthenium(II) complexes coordinated by tFMPIP (2-(trifluoromethyphenyl)-*1H*-imidazo[4,5-*f*] [1, 10] phenanthroline) can induce the apoptosis of tumor cells by regulating the expression of Bcl-2 family proteins to activate the caspase signal pathway [50]. Chalikian et al. [51] indicated that RNA can be recognized by small molecules. However, whether ruthenium(II) complexes can bind to miR-21 remains unclear. In our previous study, we studied the interaction of the polypyridine ruthenium(II) complex with the total RNA of liver cells [52]. Moreover, we demonstrated that the effect of miR-21 on phosphatase and tensin homolog deleted on the chromosome ten/protein kinase B (AKT) signaling pathway is abrogated by the arene ruthenium complexes, and the miR-21 inhibitor could enhance the antitumor capability [53].

In the current study, the binding behavior of both chiral ruthenium(II) complexes (***Λ***-1 and ***Δ*-**1, Fig. 1a) with miR-21 was first investigated. Results suggested that ***Λ***-1 and ***Δ***-1 can bind and stabilize the hairpin structure of miR-21 molecules (Fig. 1b) with obvious enantiomer selectivity. Furthermore, the expression of miR-21 could be inhibited by both isomers after enriching in the nucleus of highly metastatic human breast cancer cells through real-time images and quantitative PCR experiments.

## Results and discussion

### Recognition of Λ-1 and Δ-1 with miR-21

MiR-21, a miRNA overexpressed in almost all types of human malignancy, is involved in multiple cancer-associated processes, including proliferation, invasion and metastasis. Thus, the binding behaviors of ***Λ***-1 and ***Δ***-1 with miR-21 were investigated to clarify the interaction of chiral ruthenium(II) complex on miR-21.

**Fig. 2:**
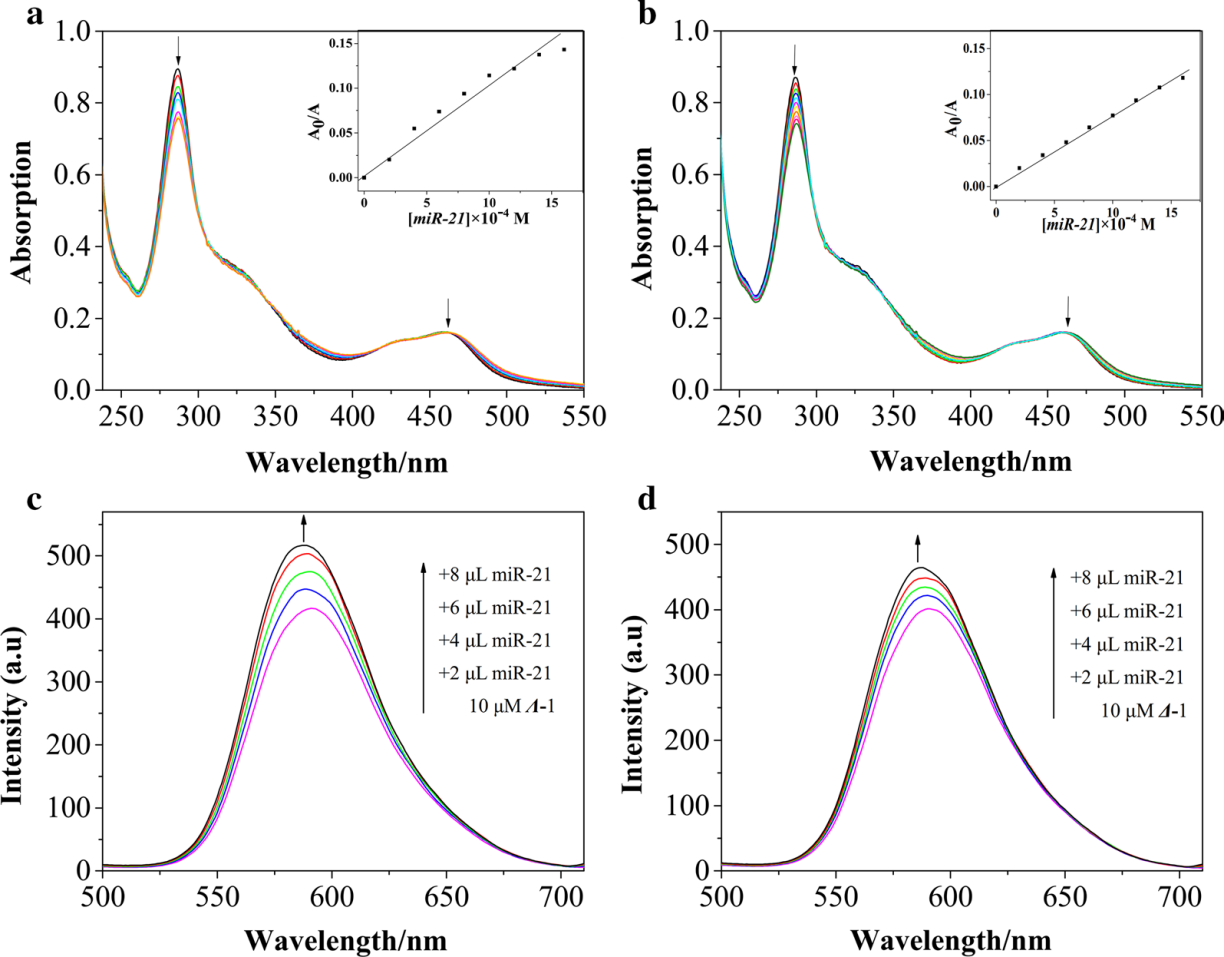
Electronic spectra of ***Λ***-1 (**a**) and ***Δ*-**1 (**b**) with and without increasing amounts of miR-21. [Ru] = 10 μM; Fluorescence emission spectra of ***Λ***-1 (**c**) and ***Δ***-1 (**d**) with and without increasing amounts of miR-21. [Ru] = 10 μM

The recognition of miR-21 by ***Λ***-1 and ***Δ***-1 was first demonstrated by electronic titration methods, which are commonly used to investigate the interaction of transition metal complexes with biological macromolecules [54]. The electronic spectra of ***Λ****/****Δ***-1 display the characterized metal-to-ligand charge transfer (MLCT) absorption with the peak at 465 nm and the characterized IL absorption with the peak at 286 nm (Fig. 2a, b). Upon the addition of miR-21 solution, apparent hypochromism occurred for both isomers because of the electronic circumstance disturbed by miR-21 molecules [55]. At [miR-21]/[Ru] = 0.083, the hypochromism values for ***Λ***-1 and ***Δ***-1 were about 15.4% and 13.2%, with the binding constant of **Λ**-1 and Δ-1 are 2.7 × 10^5^ M^−1^ and 1.14 × 10^5^ M^−1^, respectively. The characterized fluorescent for both isomers in the range of 500–700 nm underwent apparent increase in the presence of miR-21 (Fig. 2c, d). At [miR-21]/[Ru] = 0.133, the emission intensities of ***Λ***-1 and ***Δ***-1 increased to around 1.29 and 1.15 times than the original, with the intrinsic binding constant of Λ-1 and Δ-1 are 9.3 × 10^4^ M^−1^ and 1.23 × 10^4^ M^−1^, respectively. These data suggested that both isomers can bind to miR-21 in promising binding affinity, with the ***Λ***-isomer having a stronger binding affinity than the ***Δ***-isomer [56].

**Fig. 3:**
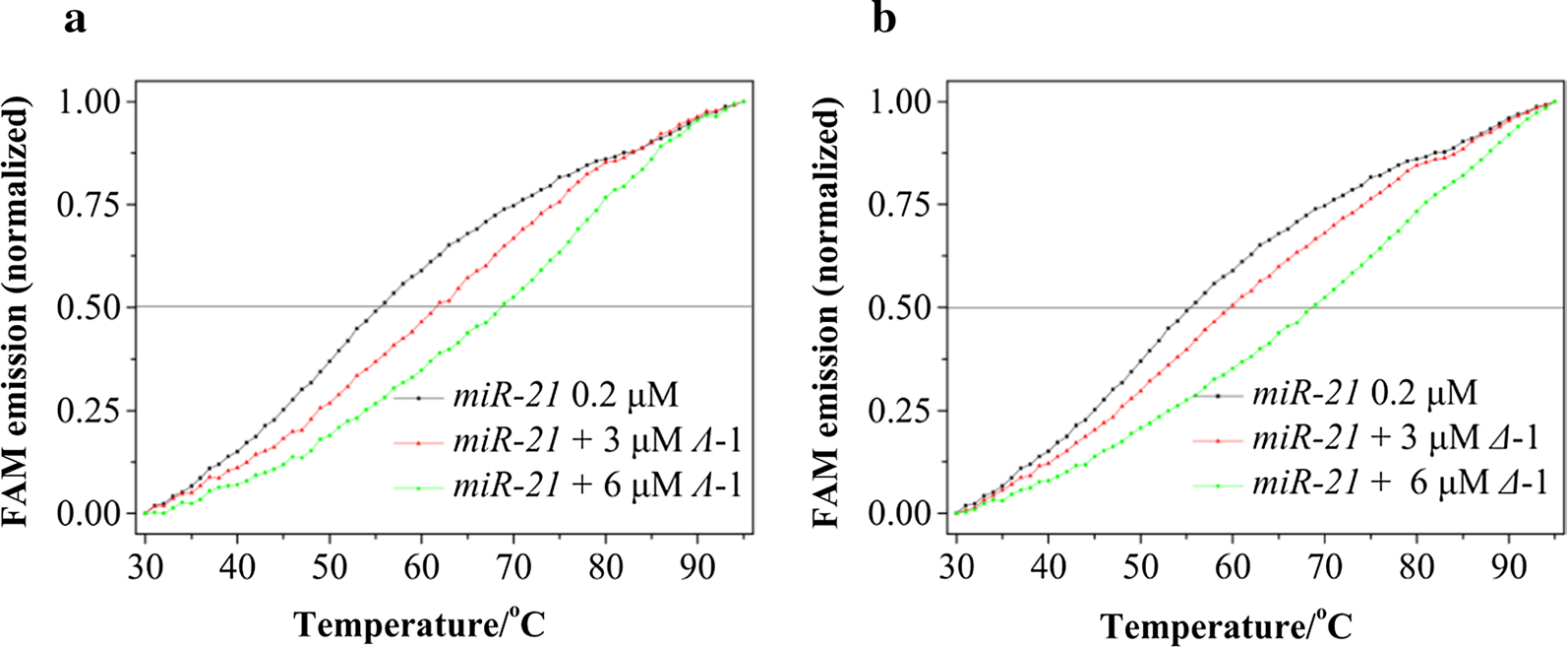
Fluorescence resonance energy transfer (FRET) melting profiles of miR-21 in the absence and presence of ***Λ*-**1 (**a**)**/*Δ***-1 (**b**). [miR-21] = 0.2 μM

The fluorescence resonance energy transfer (FRET) melting point assay was also carried out to clarify the stabilization of the structure of miR-21 in the presence of this chiral ruthenium(II) complex [57]. The melting point of miR-21 increased as the concentration of both isomers was increased. The melting point of miR-21 was about 55.1 °C (Fig. 3). When ***Λ***-1 and ***Δ***-1 were added into the solution, Tm increased in a dose-dependent manner. At [Ru] = 3.0 μM, the ΔTm values for ***Λ***-1 and ***Δ***-1 were 6.3 °C and 4.0 °C, respectively.

**Fig. 4:**
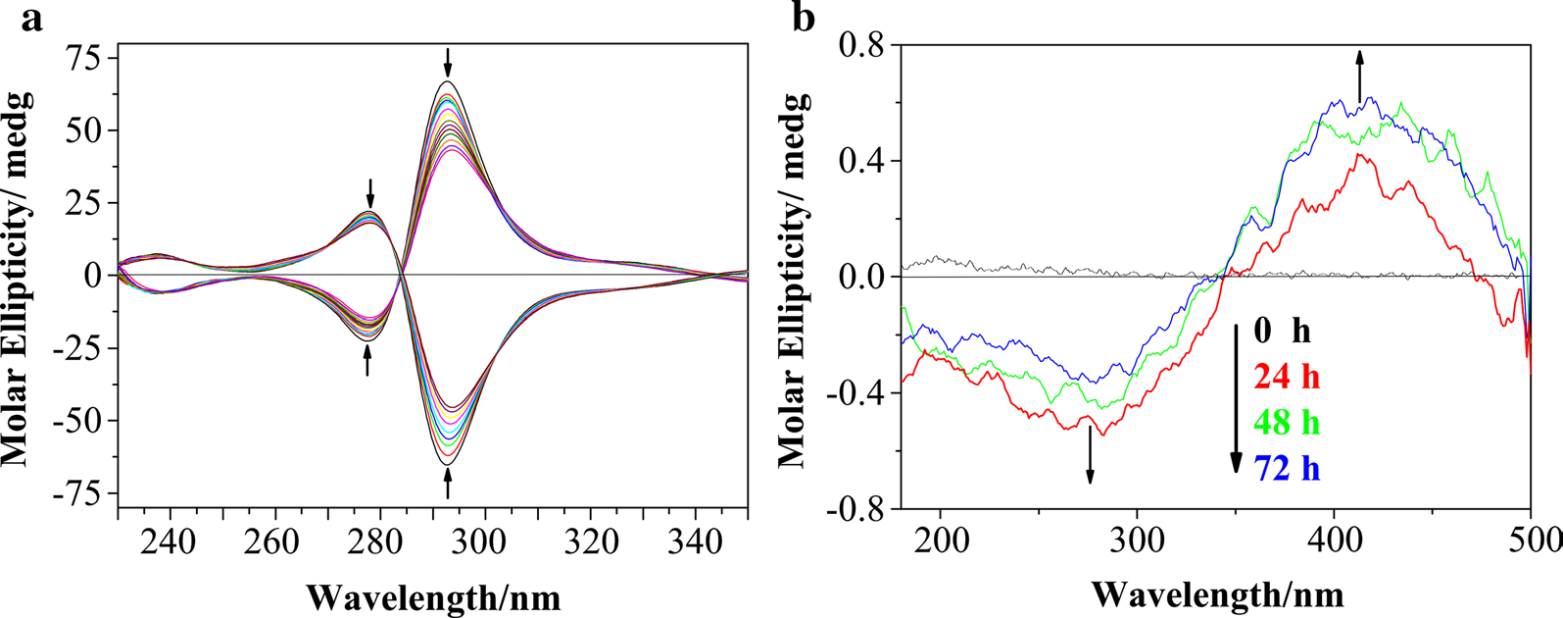
**a** CD spectra of ***Λ*-**1 and ***Δ***-1 in the absence and presence of miR-21. [Ru] = 10 μM. **b** CD spectra of racemized ruthenium(II) complex dialyzed against miR-21 at t = 0, 24, 48, and 72 h. ([Ru] = 5 μM; [miR-21] = 2.5 μM)

Furthermore, the conformation changes of both isomers in the presence of miR-21 solution were further confirmed by using circular dichroism (CD) spectroscopy [58]. When miR-21 was added into the isomer solution, the ellipticity strength of the characterized CD signal for ***Λ***-1 and ***Δ***-1 decreased obviously in a concentration-dependent manner. At [miR-21]/[Ru] = 0.183, the ellipticity strength for ***Λ***-1 and ***Δ***-1 decreased to 34.9% and 30.7%, respectively (Fig. 4a). These results indicated that the structure of both isomers can be disturbed by miR-21, and the change depends on the binding ability. Equilibrium dialysis is an important method to study the selective interaction of bio-macromolecules with small drug molecules. In general, the equilibrium dialysis was conducted at room temperature with 0.4 mL of miR-21 (25 μM) sealed in a dialysis bag and 10 mL of racemize [Ru(bpy)_2_(*p*-TEPIP)](ClO_4_)_2_ outside the bag. The CD signals of the dialyzate of racemized ruthenium(II) complex changed during dialysis. As shown in Fig. 4b, no detectable CD signal was observed before dialysis. After dialysis for 12 h, a distinct negative CD signal (300 nm) attributed to the signal of Δ-1 peaked after 24 h. These results suggested that Λ-1 exhibited greater affinity to miR21 than Δ-1.

### Expression of miR-21 regulated by Λ-1 and Δ-1

MiR-21 acts as a key regulator for cell apoptosis through a mitochondrial-dependent signal pathway. Thus, quantitative PCR (RT-qPCR) was evaluated to investigate the effect of the expression of miR-21, as shown in Fig. 2b. The levels of miR-21 markers in MDA-MB-231 cells after treatment with both isomers were analyzed.

**Fig. 5:**
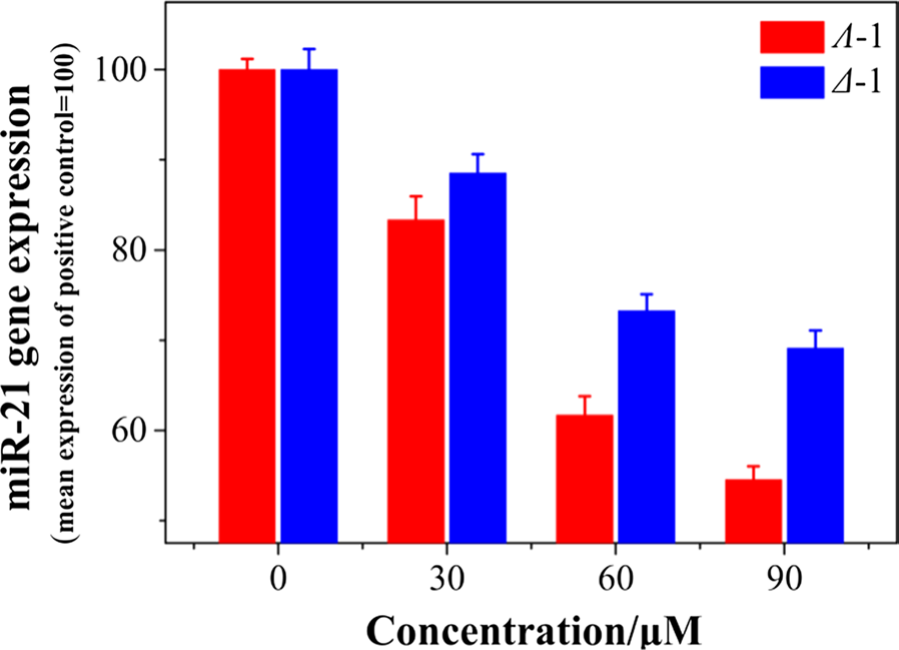
Downregulation of miR-21 after treatment with different concentration complexes in cell level tested by quantitative PCR (RT-qPCR). The amplification plot and dissociation curve of RT-qPCR of miR-21 transcript expression profiles of both isomers at various confluences are shown in Additional file 1: Figure S6

RT-qPCR showed that the expression levels of miR-21 were markedly suppressed with increasing amount of the isomers. At [Ru] = 50 μM, 45.8% and 30.7% of miR-21 gene expression (mean expression of positive control = 100) in MDA-MB-231 cells were smothered (Fig. 5). The data indicated that the expression of miR-21 could be inhibited by this type of chiral ruthenium(II) complex.

## Materials and methods

### Chemicals

All materials and solvents were obtained commercially and used without further purification unless special statement. Mature miR-21 sequence, the complementary strand 5′-UAGCUUAUCAGACUGAUGUUGA-3′, was purchased from Suzhou GenePharma Co., Ltd. F-miR-21 oligomers, the complementary strand 5′-FAM-UAGCUUAUCAGAC UGAUGUUGA-BHQ-1-3′, was also purchased from Suzhou GenePharma Co., Ltd. Both isomers **Λ/Δ**-[Ru(bpy)_2_(p-TEPIP)](ClO_4_)_2_ (**Λ/Δ-1)** were synthesized as previously described in the literature [59]. The detailed characterization data are listed in Additional file 1: Figures S1–S4, and the purity of **Λ/Δ-1** was higher than 95% as tested by HPLC (Additional file 1: Figure S5).

### Quantitative reverse transcription-PCR assay for miR-21 expression

Quantitative PCR (Q-PCR) analysis of miR-21 transcript expression profiles of both isomers at various confluences were measured using the AB 7900HT Real-Time PCR system as previously described [60] with some modifications. All conditions of this experiment were similar to Bcl-2, except miR-21 was replaced. The primers were as follows: miR-21 Forward, 5′-ACACTCCAGCTGG GTAGCTTATCAGACTGA-3′; Reverse, 5′-GTGTCG TGGAGTCGGCAATTC-3′; U6 Forward, 5′-GTGCTC GCTTCGGCAGCACATATAC-3′; Reverse, 5′ AAAAA TATGGAACGCTTCACGAATTTG-3′ (Additional file 2).

### Electronic absorption measurements

Electronic absorption spectra of the ruthenium complexes (10 μM) interacted with increasing concentration of miR21 (0, 0.0667, 0.1334, 0.2… 0.667 μM) were performed on a Shimadzu UV-2550 spectrophotometer at room temperature [61]. The absorption spectrum was recorded after mixing the ruthenium(II) complex-miR-21 solution for 5 min. The addition of miR21 repeated some times until little changes were observed in the spectra, suggesting that the binding saturation was achieved.

### Fluorescence measurements

Fluorescence experiments were carried out by inreasing miR-21 solution to the ruthenium(II) complexes. The fluorescence of both ruthenium complexes were excited at 340 nm, and the emission curve was observed from 500 to 700 nm. The fluorescence spectrum was recorded after mixing the ruthenium(II) complex-miR-21 solution for 5 min. The addition of miR21 repeated some times until little changes were observed in the spectra, suggesting that the binding saturation was achieved. Due to the dilution after each titration experiment, the concentration of the ruthenium(II) complex only slightly changed.

### Fluorescence resonance energy transfer melting point assay

The FRET melting point assay was performed to clarify the affinity of chiral ruthenium(II) complexes to bind miR-21. Fluorescence melting curves were measured by a Bio-Rad real-time PCR detection system using a total reaction excitation at 470 nm constant temperature being maintained for 30 s prior to each reading to ensure a stable value [9].

### Circular dichroism spectra measurements

Circular dichroism spectra were obtained using a Jasco J810 spectropolarimeter [62]. During the titration, a 2 μL aliquot of buffered miR-21 solution was added to each cuvette of drugs, and the solutions were mixed by repeated inversion. After the solutions were mixed for ~ 5 min, the CD spectra were recorded. For each sample, CD experiments were measured at least three times at room temperature by using a quartz cell with a path length of 1 cm. The spectra were collected at wavelengths of 200–600 nm and with a scanning speed of 50 nm/min [63]. The instrument was flushed continuously with pure evaporated nitrogen throughout the experiment.

## Conclusion

The hairpin structure of miR-21 can be selectivity recognized and stabilized by chiral ruthenium(II) complexes with alkynes (***Λ***-1 and ***Δ*-**1), and the ***Λ***-isomer exhibited stronger binding affinity than the Δ-isomer. Furthermore, both isomers can be uptaken by MDA-MB-231 cells, and the expression of miR-21 was suppressed by both isomers. Thus, these chair ruthenium(II) complexes can act as potential inhibitors by recognizing, stabilizing, and downregulating the expression of miR-21. Investigation of the detailed mechanisms between their activity and downregulated miR-21 is now in progress.

## **Supplementary information**

**Additional file 1: Figure S1.** The ESI-MS spectra of ***Λ***-1(A) and ***Δ***-1(B); Figure S2. The ^1^H NMR spectra of ***Λ***-1(A) and ***Δ***-1(B); Figure S3. The ^13^C NMR spectra of ***Λ***-1(A) and ***Δ***-1(B); Figure S4. The ^1^H ^1^H COSY spectra of ***Λ***-1(A) and ***Δ***-1(B); Figure S5. The HPLC analysis of ***Λ***-1(A) and ***Δ***-1(B); Figure S6. The amplification plot and dissociation curve of Q-PCR of miR-21 transcript expression profiles of ***Λ***-1 (A) and ***Δ***-1 (B) at various confluences.

**Additional file 2.** Raw data for RT-qPCR analysis.

## Data Availability

The datasets used and/or analysed during the current study are available from the corresponding author on reasonable request.

## Abbreviations

MiR-21: MicroRNA-21
MDA-MB-231: Human breast cancer cells
Q-PCR: Quantitative real time polymerase chain reaction
Bcl-2: B-cell lymphoma-2
Bax: B-cell lymphoma-associated x
PDCD4: Programmed cell death 4
PTEN: Phosphatase and tensin homolog deleted on chromosome ten
AKT: Protein kinase B
MLCT: Metal-to-ligand charge transfer
FRET: Fluorescence resonance energy transfer
ITC: Isothermal titration calorimetry
CD: Circular dichroism
DEPC: Diethylpyrocarbonate

## References

[1] Bergamo A, Gava B, Alessio E (2002). Ruthenium-based NAMI-A type complexes with in vivo selective metastasis reduction and in vitro invasion inhibition unrelated to cell cytotoxicity. Int J Oncol.

[2] Stevens SK, Strehle AP, Miller RL (2013). The anticancer ruthenium complex KP1019 induces DNA damage, leading to cell cycle delay and cell death in Saccharomyces cerevisiae. Mol Pharmacol.

[3] Xiangjun M, Leyva ML, Marjorie J (2009). A ruthenium-containing organometallic compound reduces tumor growth through induction of the endoplasmic reticulum stress gene CHOP. Cancer Res.

[4] Denis D, Adam SJ, Sean OB (2009). Anticancer activity of CX-3543: a direct inhibitor of rRNA biogenesis. Cancer Res.

[5] Hoelder S, Clarke PA, Workman P (2012). Discovery of small molecule cancer drugs: successes, challenges and opportunities. Mol Oncol.

[6] Cheng YC, Goz B, Minkoff M, Heindel ND (1984). Development of target-oriented anticancer drugs. Am J Clin Oncol.

[7] Dunn GP, Rinne ML, Jill W (2012). Emerging insights into the molecular and cellular basis of glioblastoma. Genes Dev.

[8] Zhang J, Yang PL, Gray NS (2009). Targeting cancer with small molecule kinase inhibitors. Nat Rev Cancer.

[9] Cheng AM, Byrom MW, Jeffrey S, Ford LP (2005). Antisense inhibition of human miRNAs and indications for an involvement of miRNA in cell growth and apoptosis. Nucleic Acids Res.

[10] Jing Z, Kuei-Chun W, Wei W (2011). MicroRNA-21 targets peroxisome proliferators-activated receptor-alpha in an autoregulatory loop to modulate flow-induced endothelial inflammation. Proc Natl Acad Sci USA.

[11] Hideo N, Min KR, Susumu T (2010). OncomiR addiction in an in vivo model of microRNA-21-induced pre-B-cell lymphoma. Nature.

[12] Yanjie L, Jiening X, Huixian L (2009). A single anti-microRNA antisense oligodeoxyribonucleotide (AMO) targeting multiple microRNAs offers an improved approach for microRNA interference. Nucleic Acids Res.

[13] Woo KS, Zhihua L, Moore PS (2010). A sensitive non-radioactive northern blot method to detect small RNAs. Nucleic Acids Res.

[14] Yue L, Cheng L, Ka-Chun W, Ke J, Zhaolei Z (2014). Inferring probabilistic miRNA–mRNA interaction signatures in cancers: a role-switch approach. Nucleic Acids Res.

[15] Bruna Karina BH, Oda JMM, Roberta LG, Carolina Batista A, Oliveira CEC, De Watanabe MAE (2014). Molecular markers for breast cancer: prediction on tumor behavior. Dis Markers.

[16] Visakorpi T (2003). The molecular genetics of prostate cancer. Urology..

[17] Judit R, Xiaohua N, Mark C (2012). A novel source for miR-21 expression through the alternative polyadenylation of VMP1 gene transcripts. Nucleic Acids Res.

[18] Sara C, Rachel C, Luebke KJ (2009). Discovering ligands for a microRNA precursor with peptoid microarrays. Nucleic Acids Res.

[19] Scott D, Bridget L, Susan F, Christine E (2006). Improved targeting of miRNA with antisense oligonucleotides. Nucleic Acids Res.

[20] Davis BN, Hilyard AC, Giorgio L, Akiko H (2008). SMAD proteins control DROSHA-mediated microRNA maturation. Nature.

[21] Leite KRM, Reis ST, Viana N (2015). Controlling reck mir21 promotes tumor cell invasion and is related to biochemical recurrence in prostate cancer. J Cancer.

[22] Joshi SR, Mclendon JM, Comer BS, Gerthoffer WT (2011). MicroRNAs-control of essential genes: implications for pulmonary vascular disease. Pulm Circ.

[23] Wu K, Li L, Li S (2015). Circulating microRNA-21 as a biomarker for the detection of various carcinomas: an updated meta-analysis based on 36 studies. Tumor Biol.

[24] Xu Y, Sun J, Xu J, Li Q, Guo Y, Zhang Q (2012). miR-21 Is a promising novel biomarker for lymph node metastasis in patients with gastric cancer. Gastroenterol Res Pract..

[25] Zhu S, Wu H, Wu F, Nie D, Sheng S, Mo YY (2008). MicroRNA-21 targets tumor suppressor genes in invasion and metastasis. Cell Res.

[26] Wickramasinghe NS, Manavalan TT, Dougherty SM, Riggs KA, Li Y, Klinge CM (2009). Estradiol downregulates miR-21 expression and increases miR-21 target gene expression in MCF-7 breast cancer cells. Nucleic Acids Res.

[27] Krichevsky AM, Gabriely G (2009). miR-21: a small multi-faceted RNA. J Cell Mol Med.

[28] Meng F, Henson R, Wehbe-Janek H, Ghoshal K, Jacob ST, Patel T (2007). MicroRNA-21 regulates expression of the PTEN tumor suppressor gene in human hepatocellular cancer. Gastroenterology.

[29] Giridhar M, George-William JN, Santoshi M (2011). Curcumin regulates miR-21 expression and inhibits invasion and metastasis in colorectal cancer. Biosci Rep.

[30] Si ML, Zhu S, Wu H, Lu Z, Wu F, Mo YY (2007). miR-21-mediated tumor growth. Oncogene..

[31] Itani S, Kunisada T, Morimoto Y (2012). MicroRNA-21 correlates with tumorigenesis in malignant peripheral nerve sheath tumor (MPNST) via programmed cell death protein 4 (PDCD4). J Cancer Res Clin Oncol.

[32] Flavie S, Marion G, Hubert L, Louis B, Pierre C (2013). Targeting miR-21 for the therapy of pancreatic cancer. Mol Ther.

[33] Shi L, Chen J, Yang J, Pan T, Zhang S, Wang ZJBR (2010). MiR-21 protected human glioblastoma U87MG cells from chemotherapeutic drug temozolomide induced apoptosis by decreasing Bax/Bcl-2 ratio and caspase-3 activity. Brain Res.

[34] Thales P, Alice S, Kosik KS (2008). MicroRNA-21 targets a network of key tumor-suppressive pathways in glioblastoma cells. Cancer Res.

[35] Lu Z, Liu M, Stribinskis V (2008). MicroRNA-21 promotes cell transformation by targeting the programmed cell death 4 gene. Oncogene.

[36] Song H, Kaiser JT, Barton JK (2012). Crystal structure of Δ-[Ru(bpy)_2_dppz]^2^+ bound to mismatched DNA reveals side-by-side metalloinsertion and intercalation. Nat Chem.

[37] Pizarro AM, Habtemariam A, Sadler PJ (2010). Activation mechanisms for organometallic anticancer complexes. Top Organomet Chem.

[38] Niyazi H, Hall JP, O’Sullivan K (2012). Crystal structures of Λ-[Ru(phen)_2_dppz]^2^+ with oligonucleotides containing TA/TA and AT/AT steps show two intercalation modes. Nature Chemistry..

[39] Pingyu Z, Jinquan W, Huaiyi H, Liping Q, Liangnian J, Hui C (2013). Chiral ruthenium(II) complexes with phenolic hydroxyl groups as dual poisons of topoisomerases I and IIα. Dalton Trans.

[40] Mei WJ, Jie L, Zheng KC (2003). Experimental and theoretical study on DNA-binding and photocleavage properties of chiral complexes Δ- and Λ-[Ru(bpy)_2_L](L = o-hpip, m-hpip and p-hpip). Dalton Trans.

[41] Marcus WL, Fredrik W, Per L, Bengt N (2002). DNA-binding of semirigid binuclear ruthenium complex Δ,Δ-[µ-(11,11′-bidppz)(phen)_4_Ru_2_]^4+^: extremely slow intercalation kinetic. J Am Chem Soc..

[42] Wiederholt K, Mclaughlin LW (1999). A 2,2″-bipyridine ligand for incorporation into oligodeoxynucleotides: synthesis, stability and fluorescence properties of ruthenium-DNA complexes. Nucleic Acids Res.

[43] Luedtke NW, Hwang JS, Eileen N, Dalia G, Moshe K, Yitzhak TJNAR (2003). The DNA and RNA specificity of eilatin Ru(II) complexes as compared to eilatin and ethidium bromide. Nucleic Acids Res.

[44] Xiang C, Jing-Heng W, Ying-Wei L, Rong Z, Hui C, Liang-Nian J (2013). Targeting telomeric G-quadruplexes with the ruthenium(II) complexes [Ru(bpy)_2_(ptpn)]^2+^ and [Ru(phen)_2_(ptpn)]^2+^. Dalton Trans.

[45] Wu Q, Zheng K, Liao S, Yang D, Li Y, Mei W (2016). Arene Ruthenium(II) complexes as low-toxicity inhibitor against the proliferation, migration, and invasion of MDA-MB-231 cells through binding and stabilizing c-myc G-quadruplex DNA. Organometallics.

[46] Fan C, Wu Q, Chen T (2014). Arene ruthenium(II) complexes induce S-phase arrest in MG-63 cells through stabilization of c-Myc G-quadruplex DNA. Medchemcommun..

[47] Chatna R, Ramune R, Linda S, Ihtshamul H, Thomas JA (2006). Dinuclear monointercalating RuII complexes that display high affinity binding to duplex and quadruplex DNA. Chem Eur J.

[48] Qian C, Wang JQ, Song CL, Wang LL, Ji LN, Chao H (2013). The induction of mitochondria-mediated apoptosis in cancer cells by ruthenium(II) asymmetric complexes. Metallomics..

[49] Vanessa P, Tanmaya J, Anna L (2012). Molecular and cellular characterization of the biological effects of ruthenium(II) complexes incorporating 2-pyridyl-2-pyrimidine-4-carboxylic acid. J Am Chem Soc.

[50] Chen T, Mei WJ, Wong YS (2010). Correction: chiral ruthenium polypyridyl complexes as mitochondria-targeted apoptosis inducers. Medchemcomm..

[51] Ghazaryan AA, Dalyan YB, Haroutiunian SG (2006). Thermodynamics of interactions of water-soluble porphyrins with RNA duplexes. J Am Chem Soc.

[52] Mei WJ, Wei XY, Liu J, Lu WG (2007). Synthesis, characterization and RNA-binding properties of a novel ruthenium(II) complex coordinated by 5-pyridine-10,15,20-triphenylporphyrin. Transition Met Chem.

[53] Wu Q, He J, Mei W, Zhang Z, Wu X, Sun F (2014). Arene ruthenium(ii) complex, a potent inhibitor against proliferation, migration and invasion of breast cancer cells, reduces stress fibers, focal adhesions and invadopodia. Metallomics..

[54] Zhao XL, Zhao H-Q, Xu XX, Li ZS, Wang K-Z (2017). Inducement and stabilization of G-quadruplex DNA by a thiophene-containing dinuclear ruthenium(II) complex. J Coord Chem.

[55] Zhao Z, Mei W, Wu X, Wang X, Wang B, Chen S (2015). Synthesis and characterization of chiral ruthenium(II) complexes Λ/Δ-[Ru(bpy)_2_(H2iip)](ClO4)_2_ as stabilizers of c-myc G-quadruplex DNA. J Coord Chem.

[56] Engman KC, Sandin P, Osborne S, Brown T (2004). DNA adopts normal B-form upon incorporation of highly fluorescent DNA base analogue tC: NMR structure and UV–Vis spectroscopy characterization. Nucleic Acids Res.

[57] Chuanqi Z, Jinsong R, Janusz G, Jerzy L, Xiaogang Q (2012). Contrasting enantioselective DNA preference: chiral helical macrocyclic lanthanide complex binding to DNA. Nucleic Acids Res.

[58] Chung-Hang L, Daniel Shiu-Hin C, Hong-Zhang H, Zhen C, Hui Y, Dik-Lung M (2012). Luminescent detection of DNA-binding proteins. Nucleic Acids Res.

[59] Zhang Z, Wang YJ, Wu Q (2014). Microwave-assisted synthesis of Ruthenium(II) complexes with trimethylsilylacetylene as inhibitors against the migration of breast cancer cells. Aust J Chem.

[60] Gray DM, Wen JD, Gray CW, Repges R, Repges C, Raabe G, Fleischhauer J (2008). Measured and calculated CD spectra of G-quartets stacked with the same or opposite polarities. Chirality..

[61] Qianqian Y, Yanan L, Chuan W (2012). Chiral ruthenium(II) polypyridyl complexes: stabilization of g-quadruplex DNA, inhibition of telomerase activity and cellular uptake. PLoS ONE.

[62] Zhang Z, Wu Q, Wu XH (2014). Ruthenium(II) complexes as apoptosis inducers by stabilizing c-myc G-quadruplex DNA. Eur J Med Chem.

[63] Zhang S, Wu Q, Zhang H (2017). Microwave-assisted synthesis of ruthenium(II) complexes with alkynes as potential inhibitor by selectively recognizing c-myc G-quadruplex DNA. J Inorg Biochem.

