# Supplementary material for: Recognizing and stabilizing miR-21 by chiral ruthenium(II) complexes
Source: BMC Chem. 2020 Apr 3;14(1):26. doi: 10.1186/s13065-020-00672-8 (PMC7119291; doi:10.1186/s13065-020-00672-8)
Supplement: Supplementary file 1 — Additional file 1: Figure S1. The ESI-MS spectra of Λ-1(A) and Δ-1(B); Figure S2. The 1H NMR spectra of Λ-1(A) and Δ-1(B); Figure S3. The 13C NMR spectra of Λ-1(A) and Δ-1(B); Figure S4. The 1H 1H COSY spectra of Λ-1(A) and Δ-1(B); Figure S5. The HPLC analysis of Λ-1(A) and Δ-1(B); Figure S6. The amplification plot and dissociation curve of Q-PCR of miR-21 transcript expression profiles of Λ-1 (A) and Δ-1 (B) at various confluences. [file 13065_2020_672_MOESM1_ESM.docx]

Additional information for

Recognizing and stabilizing miR-21 by chiral ruthenium(II) complexes

YIN FENG^1#^, JING SHU^2,3#^, LIANGZHONG YAO^1*^, YUTAO LAN^2,4*^,LIANBAO YE^2,3,5^, WENJIE MEI^2,3,5*^ and YING DING^1,2*^

^1^ The First Affiliation Hospital, Guangdong Pharmaceutical University, Guangzhou 510062, China.

^2^ Guangdong Province Engineering Center for Molecular Probe & Biomedical Imaging, Guangzhou, 510006, China.

^3^ School of Pharmacy, Guangdong Pharmaceutical University, Guangzhou, 510006, China.

^4^ School of Nursing, Guangdong Pharmaceutical University, Guangzhou, Guangdong, 510006, China.

^5^ Guangzhou key laboratory of construction and application of new drug screening model system, Guangdong Pharmaceutical University, Guangzhou, 510006, China.

Contents:

Figure S1. The ESI-MS spectra of ***Λ***-1(A) and ***Δ***-1(B);

Figure S2. The ^1^H NMR spectra of ***Λ***-1(A) and ***Δ***-1(B);

Figure S3. The ^1^H ^1^H COSY spectra of ***Λ***-1(A) and ***Δ***-1(B);

Figure S4. The HPLC analysis of ***Λ***-1(A) and ***Δ***-1(B);

Figure S5. The amplification plot(left) and dissociation curve(right) of Q-PCR of miR-21 transcript expression profiles of ***Λ***-1 (A) and ***Δ***-1 (B) at various confluences .

Figure S1. The ESI-MS spectra of ***Λ***-1(A) and ***Δ***-1(B).


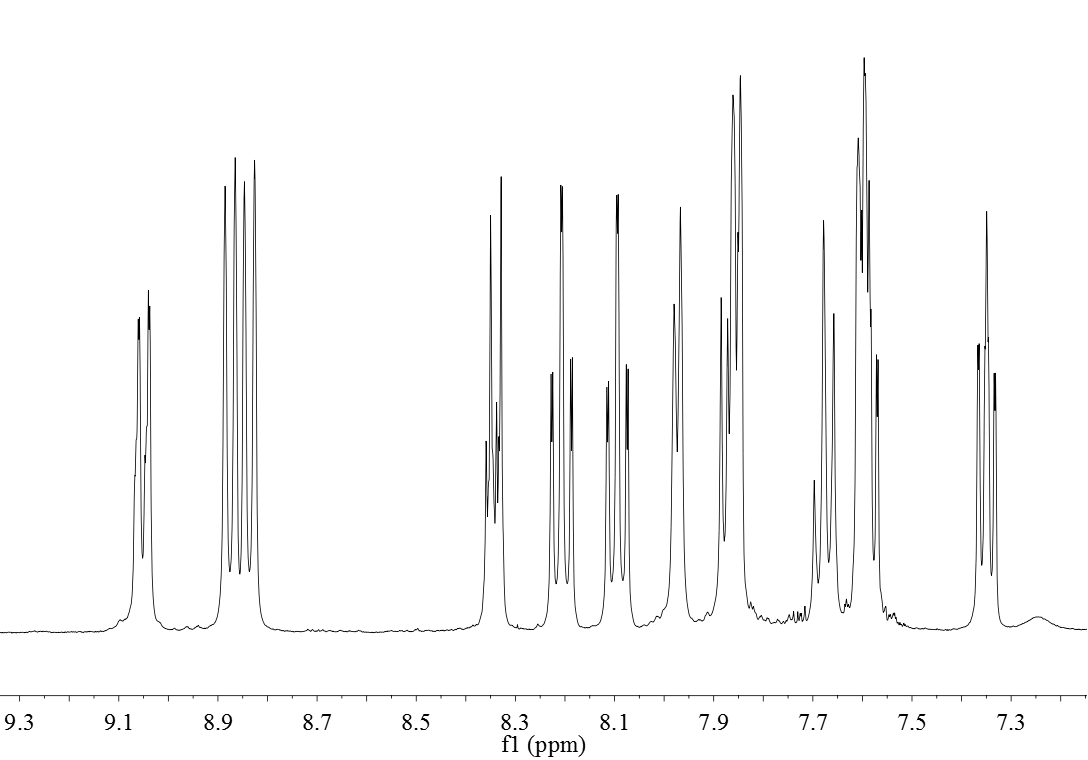


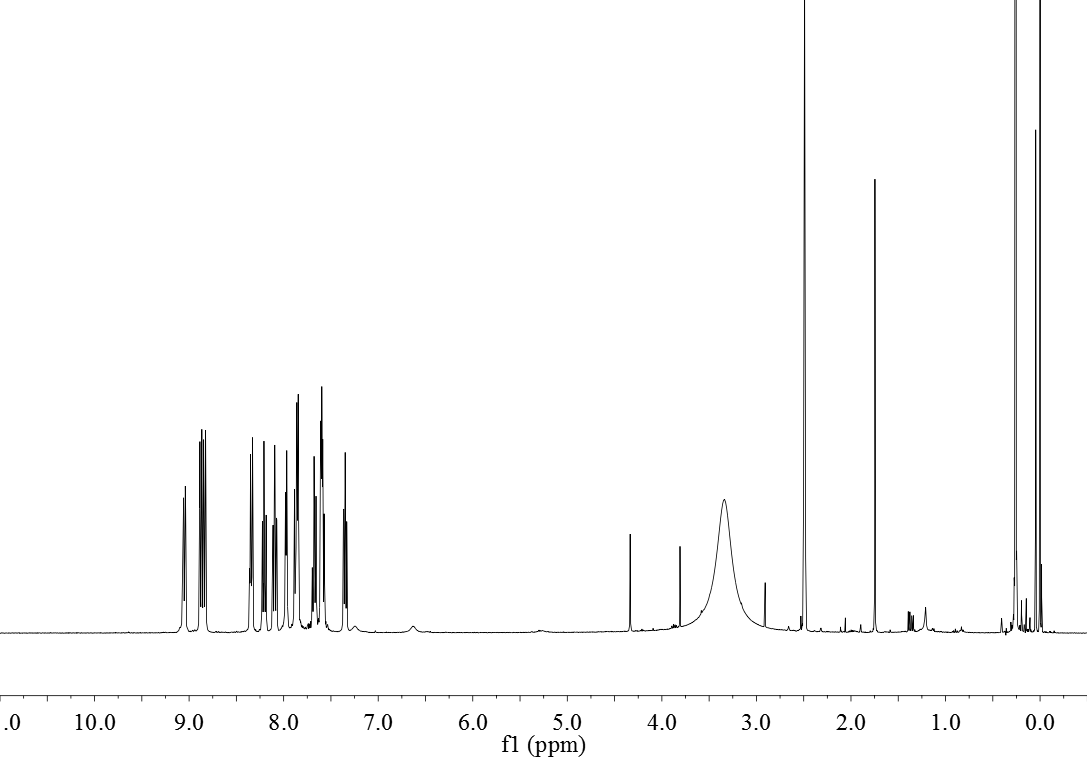


A


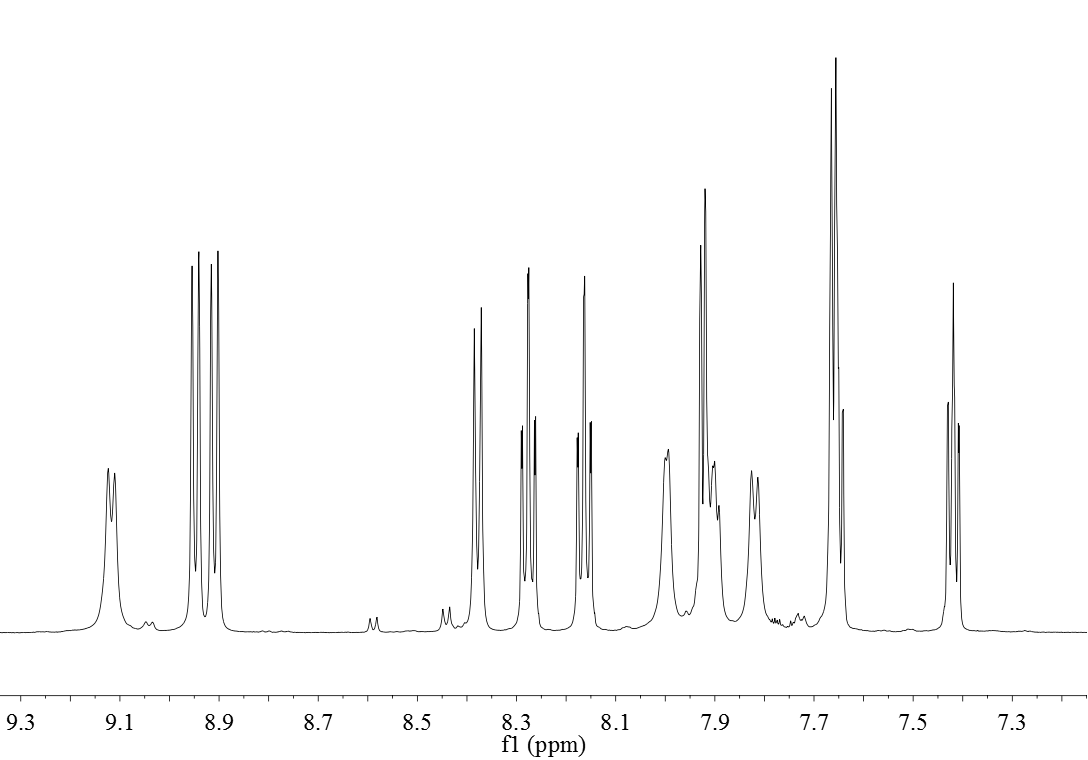


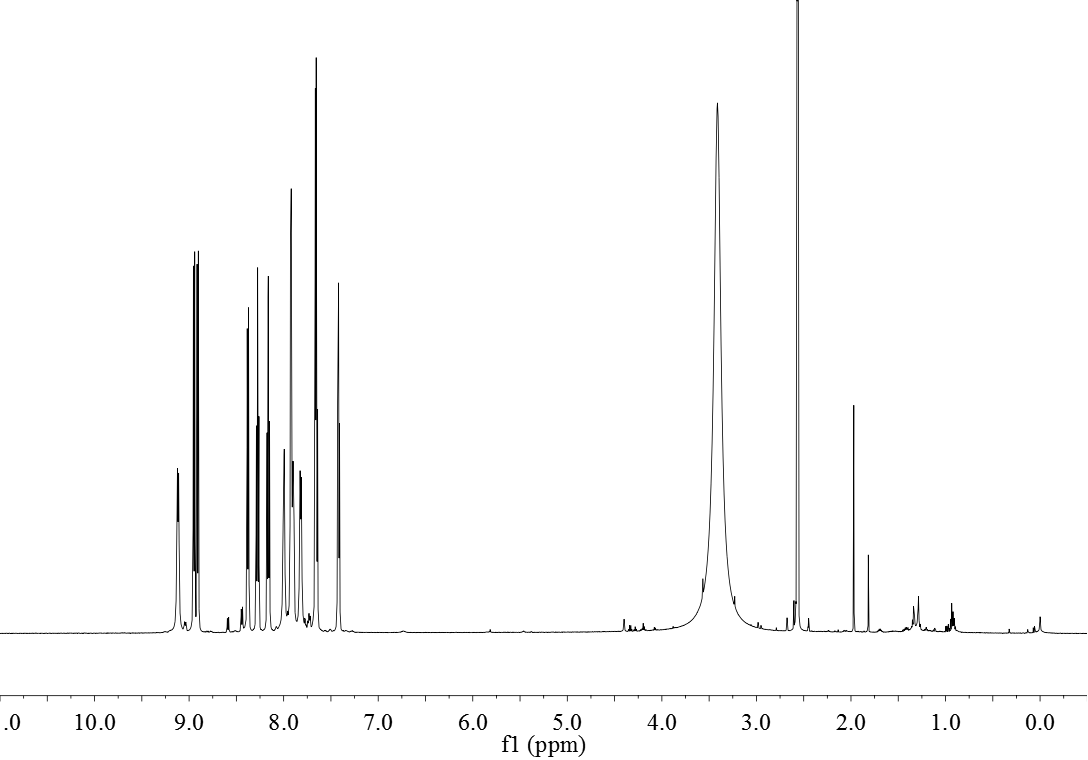


B

Figure S2. The ^1^H NMR spectra of ***Λ***-1(A) and ***Δ***-1(B).


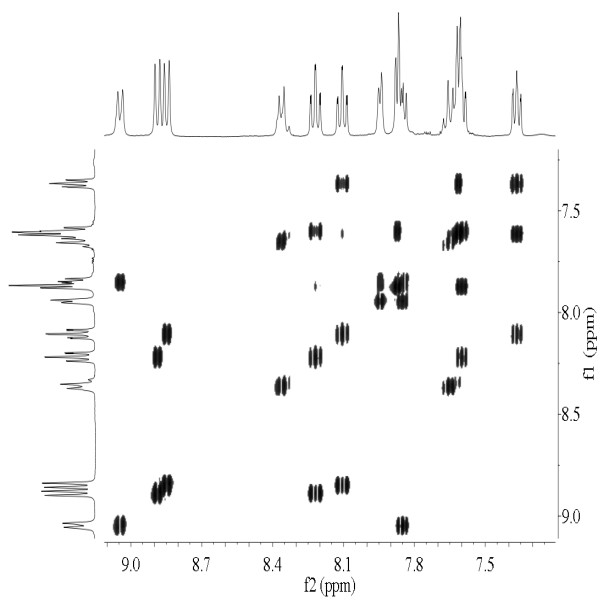


A


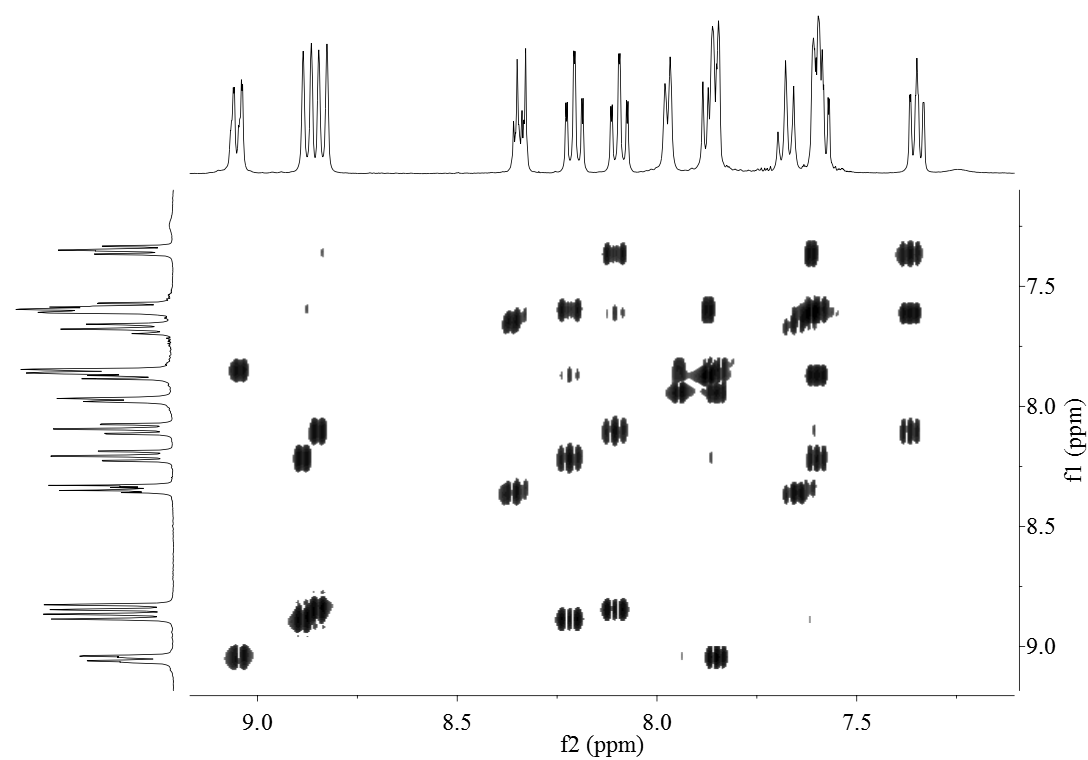


B

Figure S3. The ^13^CNM spectra of ***Λ***-1(A) and ***Δ***-1(B).

A

B

Figure S4. The HPLC analysis of ***Λ***-1(A) with purity of 94.934% and ***Δ***-1(B) with purity of 95.654%.


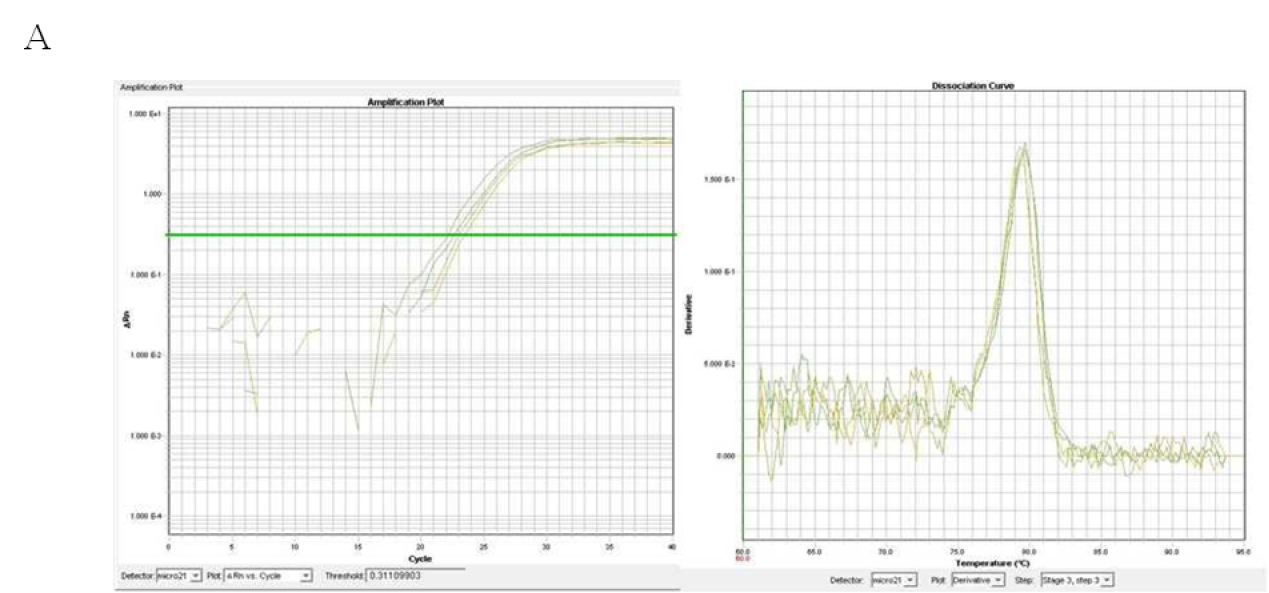


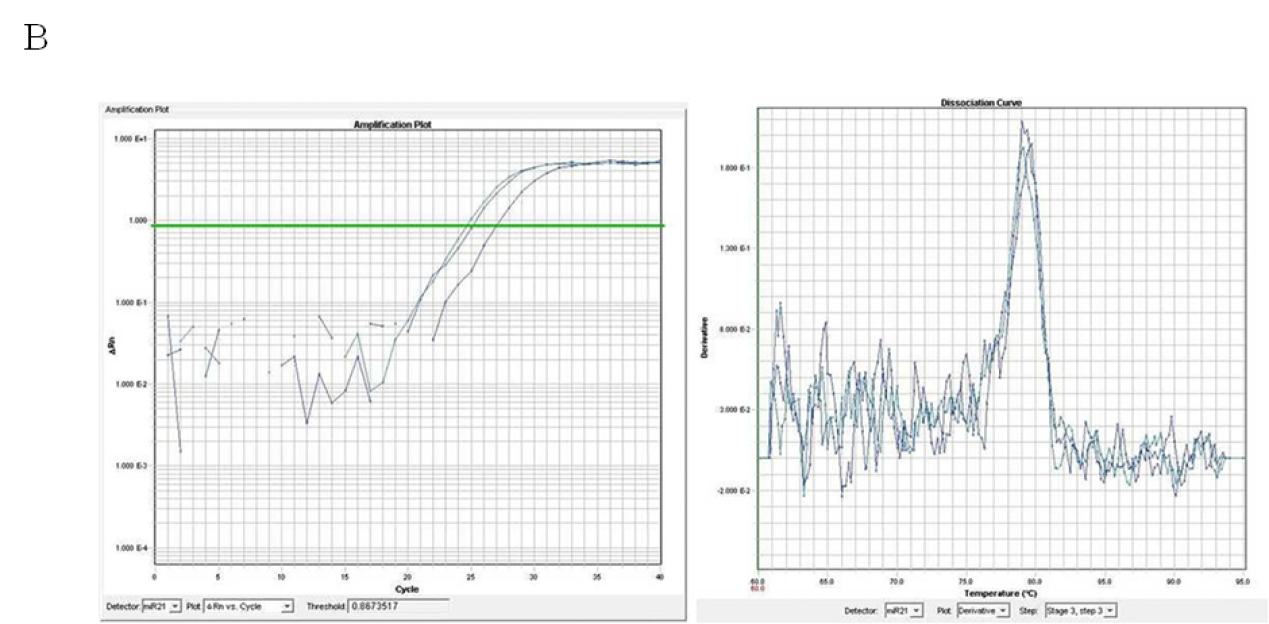


Figure S5. The amplification plot and dissociation curve of Q-PCR of miR-21 transcript expression profiles of Λ-1 (A) and Δ-1 (B) at various confluences.


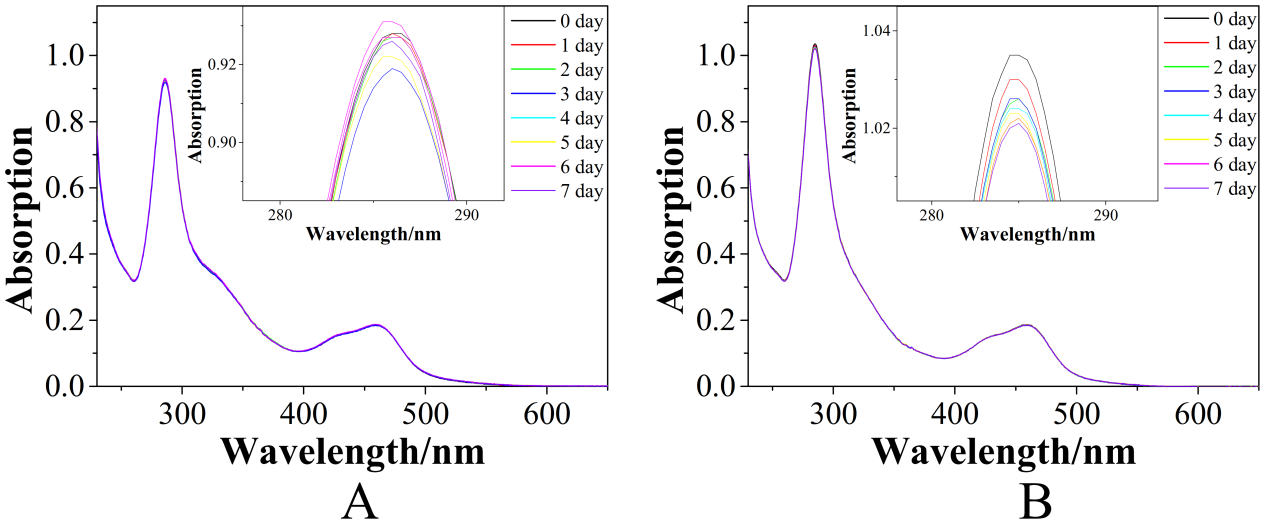


Figure S6. The stability of Λ-1 (10 μM) and Δ-1 (10 μM) in distilled water tested by UV-vis spectra in every day for 7 days.

Figure S7. Cellular localization of Λ-1 and Δ-1 in HepG2 cells. Cells were treated with Λ/Δ-1 (5 μM) for 6 h. .
